# Incidental Finding of Right-Sided Idiopathic Spontaneous Acquired Diaphragmatic Hernia

**DOI:** 10.7759/cureus.15793

**Published:** 2021-06-21

**Authors:** Zain Ali Zaidi, Sameer S Tebha, Sehrish S Sethar, Sakshi Mishra

**Affiliations:** 1 Internal Medicine, Jinnah Medical and Dental College, Karachi, PAK; 2 Neurology and Neurosurgery, Jinnah Medical and Dental College, Karachi, PAK; 3 Clinical and Translational Research, Larkin Community Hospital, Miami, USA; 4 Radiology, Jinnah Medical and Dental College, Karachi, PAK; 5 Medicine, Bangalore Medical College & Research Institute, Bengaluru, IND; 6 Otolaryngology - Head and Neck Surgery, Larkin Community Hospital, Miami, USA

**Keywords:** hiatal hernia, laparoscopic cholecystectomy, computed tomography, bochdalek hernia, case report, morgagni hernia

## Abstract

Spontaneous acquired diaphragmatic hernia is a rare finding which occurs in the absence of any trauma or surgery. Here, we present the case of an 83-year-old male who presented to the outpatient department complaining of nausea, vomiting, epigastric pain and sensation of fullness, loss of appetite, and occasional episodes of constipation, with no history of trauma or surgery. Clinical examination revealed no specific cause. A clinical diagnosis of cholelithiasis was initially suspected and confirmed by an ultrasound of the abdomen. However, considering the worsening of symptoms, a computed tomography scan revealed an incidental right-sided spontaneous diaphragmatic hernia. A subsequent laparoscopic surgery for cholecystectomy and the correction of the right-sided defect in the diaphragm was performed. spontaneous acquired diaphragmatic hernia occurring secondary to a defect on the right side of the diaphragm without any history of trauma or surgery is an extraordinary and infrequent radiological finding. Considering the challenging clinical diagnosis of such hernias, clinicians should be vigilant when patients exhibit worsening symptoms of nausea, vomiting, and gastrointestinal obstruction with or without respiratory and cardiac complications. The surgical management of such hernias is effective and secure and usually requires either an abdominal or thoracic approach and a combination of both accesses in some cases.

## Introduction

Diaphragmatic hernia is either a congenital or an acquired defect of the diaphragm muscles, pushing the abdominal content to prolapse into the thoracic cavity [1], causing several respiratory, cardiac, and abdominal complications [2]. The congenital defect is usually observed on the posterolateral side of the muscle, commonly involving the left side as the right side of the muscle is mechanically supported by the underlying liver [3]. Such diaphragmatic hernias account for more than 80-90% of the congenital hernias and are known as “Bochdalek hernias.” Other forms include the “Morgagni hernias” estimated to comprise around 2% of the congenital diaphragmatic hernias, while the central herniation makes it the rarest form of congenital diaphragmatic hernias [4].

Acquired diaphragmatic hernias mainly occur secondary to blunt trauma or penetrating injuries to the muscle [5], resulting in increased pleuroperitoneal pressure that causes an anatomic hole at the weak fusion sites of the diaphragm and upward protrusion of upper abdominal content [6]. Most often, the migrating organs are the stomach, pancreas, mesentery, spleen, and small intestines [7]. Bulging of the abdominal content into the thoracic cavity results in symptoms such as swelling, chest pain, and dyspnea, while the intestinal symptoms may manifest as abdominal pain and fullness mainly after meals, nausea, vomiting, obstructive symptoms, and even dysphagia [6].

If not secondary to trauma, acquired diaphragmatic hernias can occur as a consequence of iatrogenic defects in patients who underwent surgeries for liver transplantation [8], resection [9], or even spontaneously [5] in extremely rare cases [10].

The clinical diagnosis of such diaphragmatic hernias is highly challenging because of the heterogeneous spectrum of symptoms [2]. They are mainly diagnosed by various radiological methods, preferentially by computed tomography (CT) due to its high sensitivity and specificity [3]. Other diagnostic tools include chest radiography, ultrasonography, and magnetic resonance imaging (MRI) [11]. However, the differential diagnosis of metastasis should be considered, especially in diaphragmatic hernias with smaller defects [2].

## Case presentation

An 83-year-old male with a history of ischemic cardiomyopathy for the last three years presented to the outpatient department with major complaints of nausea, vomiting, epigastric pain, loss of appetite, abdominal fullness, and sporadic constipation for the last seven months. The patient reported that the vomiting consisted of ingested material and was usually postprandial, violent, and sudden. There was a significant loss of appetite to avoid vomiting. He also complained of localized epigastric pain with intermittent constipation leading to abdominal fullness. There was no associated fever and the patient had no symptoms of dyspnea, tachypnea, or chest pain. The patient had no significant past medical history.

On general examination, his pulse was 72 beats per minute, blood pressure was 180/110 mmHg, and SpO_2_ was 96%. On abdominal examination, his epigastrium was tender while the remaining clinical assessment was unremarkable. There were no distinct bowel sounds on auscultation. On neurological assessment, his Glasgow Coma Scale score was 15/15. There was no history of any abdominal blunt trauma or penetrating injury. The patient came to Karachi and visited Medicare Cardiac and General Hospital where he underwent several baseline investigations, including complete blood count, urea and electrolytes, prothrombin time, prostate-specific antigen test, hepatitis B and C viral screening, urinalysis, and anti-*Helicobacter pylori* antibodies, which were nonsignificant (Table 1).

**Table 1:** Baseline investigations. RBC: red blood cell; PCV: packed cell volume; MCV: mean corpuscular volume; MCH: mean corpuscular hemoglobin; WBC: white blood cell COVID-19: coronavirus disease 2019

Parameter	Result	Unit	Reference range
Hemoglobin	10.1	g/dL	M: 13.7-16.3
RBC count	3.21	10^12^/L	M: 4.5-6.5
Hematocrit (PCV)	28.9	Vol %	M: 41.9-48.7
MCV	86.1	fl	76.0-96.0
MCH	31.4	pg	26-32
WBC count	16.5	10^9^/L	4.0-11.0
Neutrophils	80	%	40-75
Lymphocytes	17	%	20-45
Eosinophils	01	%	1-6
Monocytes	02	%	2-8
Platelet count	256	10^9^/L	150-450
Creatinine	2.16	mg/dL	M: 0.9-1.3
Urea	18	mg/dL	10-50
Sodium	141	mmol/L	136-145
Potassium	3.3	mmol/L	3.5-5.1
Chloride	109	mmol/L	98-107
Bicarbonate	21	mmol/L	25-29
Prothrombin time	14.0	Seconds	12.0-16.0
International normalized ratio	1.00		
COVID-19 IgG	Negative		
COVID-19 IgM	Negative		

**Figure 1:**
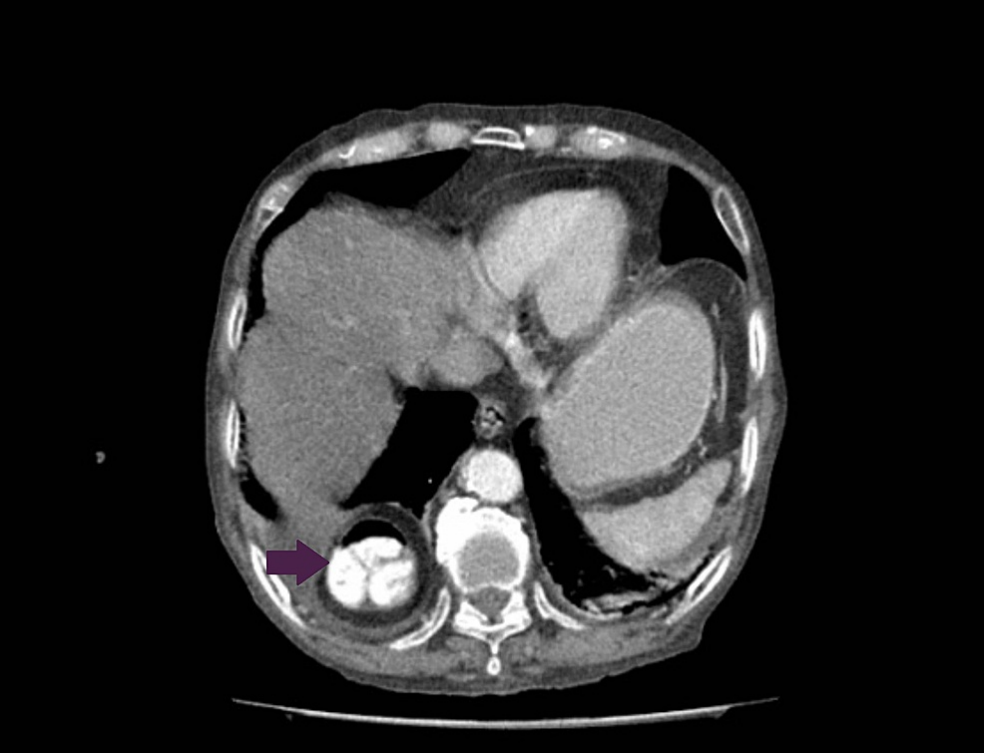
Axial view showing the herniation of contrast-filled bowel loops through the defect in the diaphragm (purple arrow).

**Figure 2:**
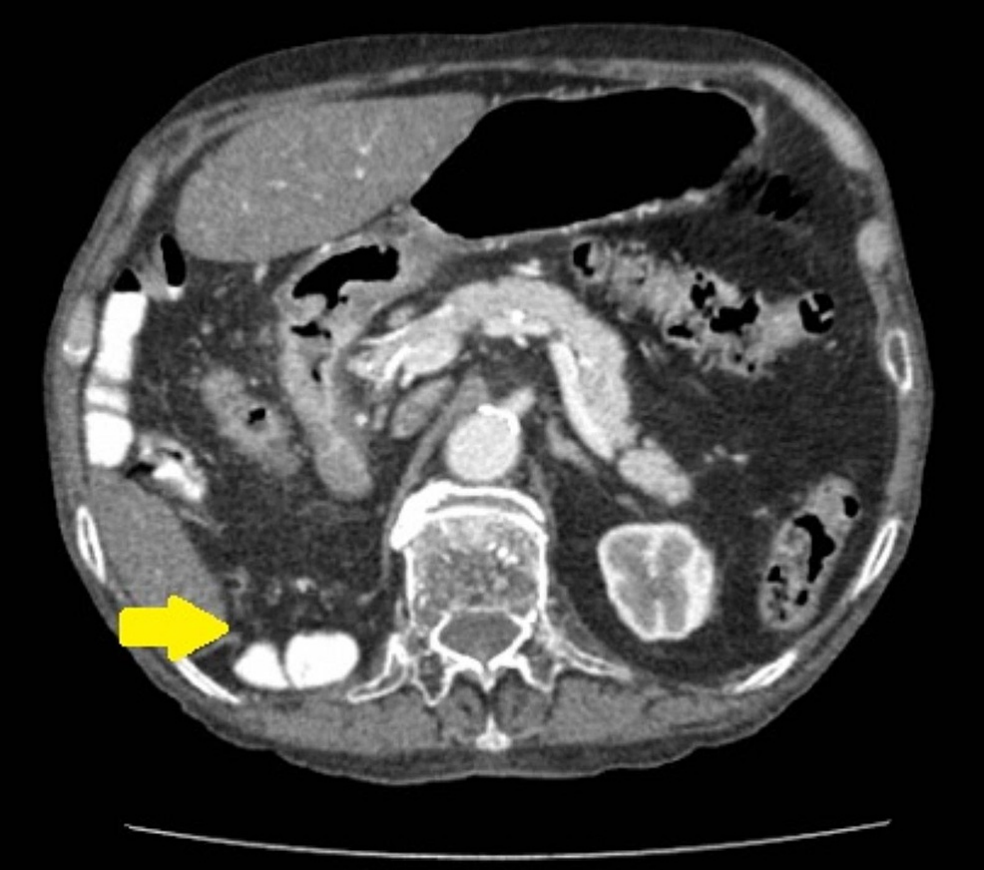
Axial view showing the defect involving the right crus of the diaphragm (yellow arrow).

Ultrasound of the abdomen confirmed the presence of cholelithiasis. Considering the progressive worsening of symptoms, a CT scan of the axial abdomen and pelvis with oral and intravascular contrast was obtained which revealed few hyperdense foci in the gallbladder lumen along with the incidental finding of a diaphragmatic hernia with a defect in the right crus, measuring 47 mm, with bowel loops and mesenteric fat herniating through the anatomical defect (Figures 1, 2). However, no signs of obstruction were observed.

The final diagnosis of a right-sided diaphragmatic hernia was made on the basis of lab investigations and imaging. As the defect had no identified underlying cause, including any history of previous trauma or surgery, the hernia was considered to be idiopathic. The patient was scheduled for a laparoscopic cholecystectomy along with the surgical correction of the defect using primary closure and nonabsorbable sutures. The hernia was successfully reduced with no complications. The patient is currently (after two months from the intervention) asymptomatic and under surveillance.

## Discussion

This is a unique case of a spontaneous diaphragmatic hernia (SDH) caused by a defect in the right crus of the diaphragm, which led to the protrusion of the bowel loop, mesenteric fat, and omentum, with no history of blunt trauma, penetrating injury, or surgery. SDHs with no underlying cause are rarely encountered with less than 30 published cases between 1956 and 2009 and less than 1% of all diaphragmatic hernias [10,12].

The two types of SDHs reported in the literature are type 1 SDH with an intact chest wall and type 2 SDH with the passage of abdominal content through the diaphragm and the chest wall. The major determinants predisposing patients to the development of such idiopathic hernias include persistent coughing, vomiting, or chronic and severe constipation [12]. According to the literature, the defect is most often present on the left crus of the diaphragm (up to 65%), while the right crus of the diaphragm is less likely to be involved due to mechanical support from the liver [3]. Hernias caused by any defect on the right side of the muscle are associated with a higher risk of morbidity and mortality [6]. Typical symptoms include nausea, vomiting, difficulty in breathing, and thoracoabdominal pain and discomfort [10]. Diaphragmatic hernias are usually diagnosed using CT with a specificity of approximately 80% and sensitivity of approximately 50% in diagnosing right-sided SDHs and a sensitivity of 78% in diagnosing left-sided SDHs, as shown by Lodhia et al [3].

This case is remarkable as right-sided defects are unusual and the heterogeneous clinical manifestation makes diagnosis challenging. However, the diagnosis of right-sided diaphragmatic hernia was made on the basis of CT axial view with oral and intravascular contrast.

The timely diagnosis of diaphragmatic hernias is necessary as they can lead to increased risk of fatality due to respiratory failure as a consequence of pressure over the lungs, reduced blood flow to the gastric and intestinal organs leading to visceral infarction, and perforations [13].

Surgical correction of the defect is considered to be a permanent cure and is usually performed using an abdominal approach with primary closure of the defect [14]. In chronic cases with delayed diagnosis, the thoracic approach is adopted to avoid complications including visceral perforation and adhesions [6]. Since in our case where the defect was not associated with signs of visceral obstruction or complications, we adopted the abdominal approach with primary closure and repaired the defect using nonabsorbable sutures. Overall, surgical correction results in a favorable outcome, with a very low rate of recurrence [5].

## Conclusions

Spontaneous acquired diaphragmatic hernia with a defect involving the right crus of the diaphragm, in the absence of any history of trauma or surgery, is a rare finding accounting for less than 1% of all diaphragmatic hernias. Considering the challenging clinical diagnosis of such hernias, clinicians should be vigilant when encountering patients with worsening symptoms of nausea, vomiting, and gastrointestinal obstruction with or without respiratory and cardiac complications. Surgical correction of such hernias is an effective and secure management option that usually requires either an abdominal or thoracic approach. However, in some cases, both approaches can be adopted.
